# Identification and analysis of glutathione S-transferase gene family in sweet potato reveal divergent *GST*-mediated networks in aboveground and underground tissues in response to abiotic stresses

**DOI:** 10.1186/s12870-017-1179-z

**Published:** 2017-11-28

**Authors:** Na Ding, Aimin Wang, Xiaojun Zhang, Yunxiang Wu, Ruyuan Wang, Huihui Cui, Rulin Huang, Yonghai Luo

**Affiliations:** 1 Plant Functional Genomics, School of Life Sciences, Jiangsu Normal University, 101 Shanghai Road, Tongshan New District, Xuzhou City, Jiangsu Province 221116 China; 2 Center for Molecular Cell and Systems Biology, College of Life Sciences, Fujian Agriculture and Forestry University, Fuzhou City, Fujian Province 350002 China

**Keywords:** Sweet potato, Glutathione S-transferase, Evolutionary diversification, Regulatory mechanism, Abiotic stresses

## Abstract

**Background:**

Sweet potato, a hexaploid species lacking a reference genome, is one of the most important crops in many developing countries, where abiotic stresses are a primary cause of reduction of crop yield. Glutathione S-transferases (*GSTs*) are multifunctional enzymes that play important roles in oxidative stress tolerance and cellular detoxification.

**Results:**

A total of 42 putative full-length *GST* genes were identified from two local transcriptome databases and validated by molecular cloning and Sanger sequencing. Sequence and intraspecific phylogenetic analyses revealed extensive differentiation in their coding sequences and divided them into eight subfamilies. Interspecific phylogenetic and comparative analyses indicated that most examined *GST* paralogs might originate and diverge before the speciation of sweet potato. Results from large-scale RNA-seq and quantitative real-time PCR experiments exhibited extensive variation in gene-expression profiles across different tissues and varieties, which implied strong evolutionary divergence in their gene-expression regulation. Moreover, we performed five manipulated stress experiments and uncovered highly divergent stress-response patterns of sweet potato *GST* genes in aboveground and underground tissues.

**Conclusions:**

Our study identified a large number of sweet potato *GST* genes, systematically investigated their evolutionary diversification, and provides new insights into the *GST*-mediated stress-response mechanisms in this worldwide crop.

**Electronic supplementary material:**

The online version of this article (DOI: 10.1186/s12870-017-1179-z) contains supplementary material, which is available to authorized users.

## Background

Gene duplication is one of central research themes in evolutionary biology through which new genetic materials for phenotypic innovations are generated. After duplication, the duplicated genes may functionally diversify in protein property and/or spatiotemporal gene-expression pattern, and eventually lead to distinct evolutionary consequences: non-functionalization, subfunctionalization, or neofunctionalization [1, 2]. A contemporary gene family in a specific species represents a set of extant genes derived from a single ancestor, as an evolutionary consequence of whole genome and/or gene duplications. Systematic investigation of a gene family, in divergence of both protein-coding sequence and gene-expression profiles, would advance our understandings towards its origin and evolution and provide important insights into gene function and application [3, 4].

Glutathione S-transferases (GSTs, EC 2.5.1.18) are a family of multifunctional dimeric enzymes, which widely function in cellular detoxification of xenobiotic and endobiotic compounds by conjugating the tripeptide glutathione (GSH; γ-L-glutamyl-L-cysteinyl-L-glycine) to various substrates [5]. *GST* genes have been ubiquitously found in eukaryotes and prokaryotes, and well-studied across plants, animals, fungi, and bacteria [6]. In higher plants, *GSTs* have been classified into eight typical subfamilies, including Phi (GSTF), Tau (GSTU), Lambda (GSTL), dehydroascorbate reductase (DHAR), Theta (GSTT), Zeta (GSTZ), elongation factor 1 gamma (EF1Bγ), and tetrachlorohydroquinone dehalogenase (TCHQD) [7, 8]. Amongst these subfamilies, Phi, Tau, Lambda, and DHAR are plant-specific [5]. A typical GST protein contains two conserved active sites: one is a GSH-binding site (G-site) in the N-terminal domain, and the other is a C-terminal co-substrate-binding domain (H-site). G-site is specific for GSH and mainly affects the catalytic function, whereas the H-site contributes to the conjunction of specific substrate [8, 9].

It has been functionally demonstrated that plant *GST* genes are widely involved in the detoxification of herbicides, as well as in response to biotic and abiotic stresses [10]. *GST* genes could respond to a wide range of stress treatments such as ozone, hydrogen peroxide, plant hormone, heavy metal, heat shock, wounding, and dehydration [11–13]. Among the eight *GST* subfamilies, the functions of Tau and Phi subfamily members are the most widely studied. For example, the expression of *AtGSTU19* could be induced in the arid environment [14–16], and *AtGSTF10* is involved in salt stress and BAK1-mediated spontaneous cell death signaling pathway [17]. In addition, genes in these two subfamilies are involved in the transport and metabolism of secondary compounds [18–20]. For example, the maize *Bz2* gene, the petunia *An9* gene, and the *Arabidopsis TT19* gene function in anthocyanin transport and vacuolar sequestration [19, 21, 22]. *GST* genes in other subfamilies are also multifunctional: some *GSTs* in the Zeta subfamily are involved in tyrosine metabolism [23, 24], some in the DHAR subfamily could catalyze the metabolism of ascorbic acid [8, 25], some in the Lambda subfamily can be used as antioxidant and selectively bound to flavonol [26], and members of the EF1Bγ subfamily mainly function as glutathione peroxidases, which protect cells from interference and damage by oxide [27, 28].

Sweet potato [*Ipomoea batatas* (L.) Lam.] is one of the most important crops in the world because it provides an indispensable caloric source for human beings, especially those living in Sub-Saharan Africa and East Asia [29]. Because sweet potato can adapt and grow well in diverse harsh environments, it ensures food supply and safety in developing countries. However, advances in fundamental research for this outcrossing hexaploid crop (2n = 6× = 90) are highly limited because of its complex genetic composition, which has been thought to experience multiple whole-genome duplications during speciation [30–32]. To date, no high-quality reference genome sequence for sweet potato is available to date. Therefore, investigations of genome-wide gene duplications and their evolution in sweet potato remain a challenge. Whole transcriptome sequencing (i.e., RNA-seq) provides a valuable alternative to whole genome sequencing for gene mining and functional characterization [33, 34]. In particular, the third-generation sequencing technologies have enabled us to obtain long-read or full-length transcriptomes, which allows collection of large-scale long-read transcripts with complete coding sequences and characterization of gene families [35–38]. In the present study, we identified 42 putative full-length and 19 partial *GST* genes from our high-quality transcriptome databases in sweet potato and investigated their divergence in coding sequences, gene-expression profiles, and biological functions in response to multiple abiotic stresses. Our study serves as the first case involving the characterization of a transcriptome-wide gene family in sweet potato, which is a genetically complex organism lacking high-quality reference genome sequences. Our results reveal new insights into distinct regulatory mechanisms in aboveground and underground tissues in *GST*-mediated response to abiotic stresses in sweet potato.

## Methods

### Generation of high-quality transcriptome databases in sweet potato by second- and third-generation RNA sequencing technologies

Previously, we reported 53,861 high-quality long-read transcripts for sweet potato, which were generated by a combination of Illumina second-generation and PacBio third-generation sequencing technologies [39]. In this study, we further assembled the obtained Illumina second-generation reads together with 53,861 long-read transcripts to generate a combined transcriptome database (named as DB12; transcript number: 200,752). DB12 was generated from a single variety of *Xushu18*, an elite sweet potato variety in China, with the aim of reducing the transcriptome complexity and improving the accuracy of the transcript assembly. In another ongoing project, we sequenced the transcriptomes of mature tuberous root of each of 77 sweet potato varieties (36 purple-flesh and 41 non-purple-flesh) using the Illumina second-generation sequencing technology. All Illumina short reads from the 77 varieties were pooled for a transcriptome assembly (namely, DB77), which generated 305,505 transcripts. The transcriptome databases of DB12 and DB77 are available upon request. Sampled tissues that were used for the generation of DB12 and DB77 were illustrated in Additional file 1: Figure S1.

### Identification of *GST* genes from the transcriptome databases DB12 and DB77

We performed local BLAST and domain search for genes containing *GST* N-terminal and C-terminal domain in the transcriptome databases DB77 and DB12. First, we retrieved *Arabidopsis GST* protein sequences from website (http://www.arabidopsis.org /). The obtained *Arabidopsis GST* protein sequences were used as the query to perform BLAST searches against DB12 and DB77. A cut-off E-value (≤ e^−3^) was applied to filter the homologous transcripts. Secondly, the obtained transcript sequences from DB12 and DB77 databases were translated and analyzed by the PFAM program (http://pfam.xfam.org) to examine the presence of the *GST* domains. Furthermore, we removed the transcripts encoding short proteins with less than 120 amino acids and confirmed the presence of *GST* domains by analyzing the deduced proteins of filtered transcripts in the NCBI Conserved Domain Database (CDD, http://www.ncbi.nlm.nih.gov/Structure/cdd/wrpsb.cgi?). The following parameters were used in the CDD analysis: E-value, 0.01; maximum number of hits, 500; and the result mode, Concise. The transcripts which did not contain a complete *GST* N-terminal or C-terminal domain in CDD analysis were eliminated. Finally, we removed one redundant sequence if two transcripts had the identity of amino acids equal to or larger than 97% and obtained a final gene list. The pairwise identity matrix of 43 full-length *GSTs* was generated by the software BioEdit [40].

### Molecular cloning and Sanger sequencing of *GST* genes in a sweet potato variety

A pooled sample (including 8 tissues of shoot, young leaf, mature leaf, stem, fibrous root, initial tuberous toot, expanding tuberous root, and mature tuberous root) was collected from a single sweet potato variety (*Nanzishu8*) that was randomly selected from DB77 varieties. Total RNA was isolated from the pooled sample using TRIzol and cDNA was synthesized by reverse transcription Kit (ProbeGene, China). To clone the transcriptome-derived *GST* genes, gene-specific primers were designed used for PCR amplification using the synthesized cDNA as templates (Additional file 2: Table S1). Amplified fragments were cloned into the vector pUC57 and subjected to Sanger sequencing. The obtained sequences were compared to the corresponding transcripts obtained from the transcriptome databases and the polymorphism data are summarized in Table 1.

**Table 1:** Comparison of putative coding sequences of 43 *GST* genes obtained from transcriptomes (CDS1) and laboratory cloning (CDS2)

Gene name	Length of CDS1 (bp)	Length of CDS2 (bp)	△Length (bp)	Type of Polymorphism	Alignment length (bp)	Identical (bp)	Identity (%)
IbDHARl	642	642	0	SNP	642	638	99.38
IbDHAR2	813	813	0	SNP	813	808	99.39
IbEFlBγl	1260	1270	10	SNP & InDel	1272	1245	97.88
IbEFlBγ2	1260	1260	0	SNP	1260	1253	99.44
IbEF1Bγ3	1293	1260	−33	SNP & InDel	1261	1155	91.59
IbGSTFl	675	675	0	SNP	675	668	98.96
IbGSTF2	642	739	97	SNP & InDel	647	264	40.80
IbGSTF3	645	643	−2	SNP	643	638	99.22
IbGSTLl	711	711	0	SNP	711	710	99.86
IbGSTL2	705	705	0	SNP	705	694	98.44
IbGSTL3	810	810	0	SNP	810	804	99.26
IbGSTTl	708	708	0	SNP	708	691	97.60
IbGSTT2	708	708	0	SNP	708	700	98.87
IbGSTUl	675	673	−2	SNP	673	667	99.11
IbGSTU2	666	666	0	N.A.	666	666	100.00
IbGSTU3	660	661	1	SNP & InDel	661	613	92.74
IbGSTU4	690	690	0	N.A.	690	690	100.00
IbGSTU5	684	684	0	SNP	684	679	99.27
IbGSTU6	672	663	−9	SNP	653	640	98.01
IbGSTU7	714	728	14	SNP & InDel	729	688	94.38
IbGSTU8	672	663	−9	SNP	663	650	98.04
IbGSTU9	714	714	0	SNP	714	707	99.02
IbGSTUlO	660	738	78	SNP & InDel	738	652	88.35
IbGSTUll	684	661	−23	SNP & InDel	651	616	94.62
IbGSTUl2	675	675	0	SNP	675	673	99.70
IbGSTUl3	672	672	0	SNP	672	658	97.92
IbGSTUl4	672	672	0	SNP	672	637	94.79
IbGSTUl5	687	689	2	SNP & InDel	689	674	97.82
IbGSTUl6	672	660	−12	SNP	660	646	97.88
IbGSTUl7	669	669	0	SNP	669	656	98.06
IbGSTUl8	666	663	−3	SNP	663	659	99.40
IbGSTUl9	729	714	−15	SNP & InDel	713	691	96.91
IbGSTU2O	678	673	−5	SNP	673	664	98.66
IbGSTU2l	684	684	0	SNP	684	676	98.83
IbGSTU22	687	687	0	SNP	687	667	97.09
IbGSTU23	702	702	0	SNP	702	699	99.57
IbGSTU24	669	665	−4	SNP	665	657	98.80
IbGSTU25	687	680	−7	SNP	680	671	98.68
IbGSTU26	744	837	93	SNP & InDel	837	739	88.29
IbGSTU27	672	662	−10	SNP & InDel	665	633	95.19
IbGSTZl	900	894	−6	SNP & InDel	900	876	97.33
IbGSTZ2	672	672	0	SNP	672	668	99.41
IbGSTZ3	672	666	−6	SNP & InDel	666	659	98.95

Note: *SNP* single nucleotide polymorphism, *InDel* insertion or deletion

### Construction of phylogenetic trees and motif analysis

The protein sequences of identified sweet potato *GSTs* were aligned and phylogenetic trees were created using MEGA 7.0 (Molecular Evolutionary Genetics Analysis) program [41]. Alignments were performed using the Muscle program with default parameters, and the results were then subjected to construct unrooted phylogenetic trees using both the Maximum Likelihood (ML) method and the Neighbor-Joining (NJ) method, where the bootstrap analyses were carried out with 1000 replicates. The online MEME program (http://meme-suite.org/) was used for motif analysis. We set the maximum number of motifs to ten and other parameters were set to default.

### Analysis of gene-expression profiles

First, we investigated the variation of gene-expression profiles in the tuberous roots of the DB77 varieties. For each *GST* gene, representative transcripts in DB77 were identified and the FPKM values of representative transcripts were extracted for a clustering analysis using the Cluster 3.0 program. The parameters of clustering analysis were as follows: all of the data were adjusted by log transformation and hierarchical clustering analysis was chosen as calculating method and complete linkage as clustering method. At last, the heat map was shown by Java TreeView.

Second, we surveyed the expression pattern of the *GST* genes in 8 different tissues of one purple-flesh (*Xuzi3*) and one non-purple-flesh (*Yan252*) sweet potato variety. The 8 tissues included shoots, young leaves, mature leaves, stems, fibrous roots, initial tuberous roots, expanding tuberous roots, and mature tuberous roots (Additional file 1: Figure S1). High-throughput RNA sequencing was performed and a transcriptome database (named as DB16) was assembled from pooled RNA-seq data of 16 samples. Representative transcripts of *GST* genes from DB16 were identified and FPKM values of representative transcripts were extracted from DB16 for a clustering analysis using the Cluster 3.0 program. In both cases, we determined the representative transcripts in a database by BLASTn search with following criteria: coverage of the first alignment larger than 40% of the investigated gene and identity of the aligned sequences larger than 97%.

### Quantitative RT-PCR (RT-qPCR) experiments

To validate the *GST* gene-expression profiles observed in the transcriptomic experiments described above, we performed RT-qPCR analysis of 9 *GST* genes using the same tissue samples in DB16. The examined genes and primer sequences are listed in Additional file 2: Table S1. Total RNA was extracted using TRIzol reagents and the provided protocol (Invitrogen, USA). For each sample, three technical replicates of RT-qPCR were done. The expression of each gene in different samples was normalized with the expression of an internal control gene, *ARF*, to ensure the equal amount of cDNA used for individual reactions. The mRNA levels for each gene in different tissue samples were calculated using the ΔΔCT method. The relative gene expression levels in 16 tissue samples were further normalized with the expression in shoot of non-purple-flesh sweet potato.

### Response of *GST* genes to abiotic stresses

To investigate the expression patterns of *GST* genes under normal growth condition and abiotic stresses, shoot cuttings of sweet potato were cultivated in 1‰ Hoagland’s hydroponic medium for 7 days and then transferred to hydroponic boxes containing 5.0% hydrogen peroxide (H_2_O_2_), 200 μM cupric sulfate (CuSO_4_), 40 μM arsenic solution (As_2_O_3_), 1.5 mM cadmium carbonate (CdCO_3_) and 2 mM zinc vitriol (ZnSO4·7H_2_O), respectively. Cultivation in 1‰ Hoagland’s hydroponic medium was used as control. Each treatment consisted of eight duplicates of shoot cuttings (classified into two subgroups, each group had four duplicates). After 48 h, the aboveground (including shoots, young leaves, and mature leaves) and underground (adventitious roots) tissues were collected, respectively, from each subgroup. All samples were frozen immediately with liquid nitrogen and stocked in a − 80 °C freezer. Total RNA extraction, cDNA synthesis, and quantitative real-time PCR were performed as described above.

## Results

### Identification and validation of transcriptome-wide *GST* genes in sweet potato

We searched the two transcriptome databases (DB12 and DB77) using BLAST to identify candidate *GST* transcripts. The presence of conserved *GST* N-terminal domain (i.e., PFAM domain PF02798) in each transcript was confirmed in the NCBI Conserved Domain Database, and redundant transcripts were removed. A total of 62 putative non-redundant *GST* genes were identified (Additional file 3: Table S2). Domain structure analysis suggested that 43 of these had complete N- and C- terminal domains (i.e., full-length *GST* genes) and 19 had only a N-terminal or C-terminal domain (i.e., partial *GST* genes). Sequence comparisons indicated that the pairwise identity of the coding sequences (CDS) of the full-length *GSTs* ranged from 0.127 to 0.979, whereas the pairwise identity of the deduced amino acids ranged from 0.050 to 0.951 (Additional file 4: Table S3).

Erroneous transcripts could be generated in high-throughput transcriptome sequencing projects due to assembly errors. To validate the transcriptome-derived sequences, we designed gene-specific primers and cloned the predicted coding sequences of all 43 full-length *GST* genes from a single sweet potato variety (*Nanzishu8*) that were randomly picked up from the DB77 varieties. cDNA sequences of all 43 *GST* genes were successfully cloned from *Nanzishu8* and all but one (i.e., *IbGSTF2*) sequences were highly homologous to the corresponding *GSTs* that were identified in the transcriptome databases (Table 1). In contrast to the transcriptome-derived *IbGSTF2* sequence, the cloned fragment shared only 40.80% identity. This was likely attributable to incorrect transcriptome assembly or molecular cloning. We also found relatively low identity between transcriptome-derived and cloned sequences of *IbGSTU10*, which was actually due to the existence of a 78-bp insertion in the cloned copy. A similar situation was found for *IbGSTU26*. The other 40 *GST* sequences cloned from *Nanzishu8* shared high identity (i.e., > 90%) with those derived from our transcriptome databases (Table 1). Considering the presence of genetic variation among sweet potato varieties, we concluded that except for *IbGSTF2*, the other 42 *GST* genes identified in our transcriptome databases were indeed present in the sweet potato genome.

### Phylogenetic and comparative analyses of *GST* genes in and beyond the sweet potato species

**Fig. 1:**
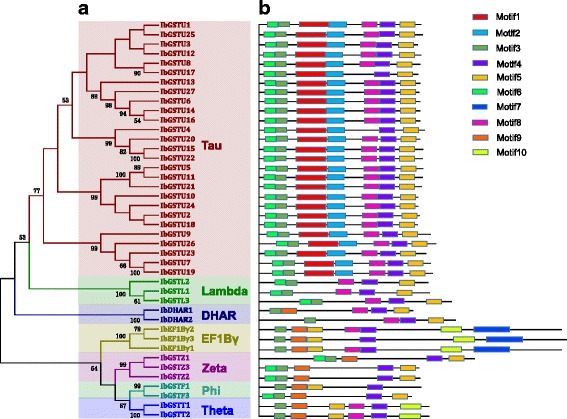
Phylogenetic relationships, gene-expression profiles and putative motifs of 42 sweet potato GST proteins. **a** Multiple alignments of amino acids of 42 full-length GST genes from sweet potato were executed by Muscle Program and the phylogenetic tree was constructed using MEGA 7.0 by the Maximum Likelihood (ML) method. The percentage bootstrap scores which larger than 50 are indicated on the nodes. The major phylogenetic subfamilies are illustrated as different color branches. **b** Schematic representations of the conserved motifs in the GST proteins predicted by MEME search. Black lines represent non-conserved sequences

**Fig. 2:**
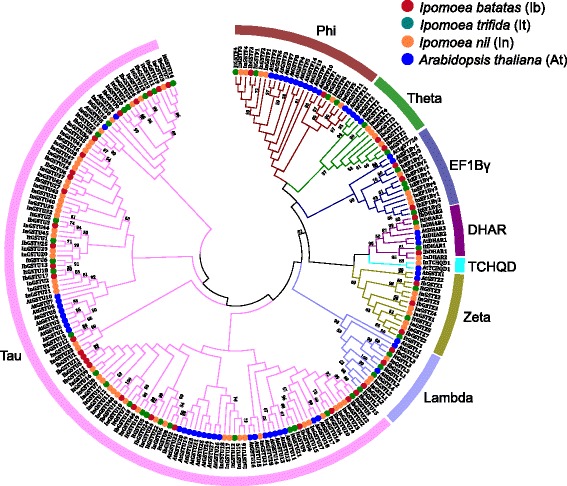
Phylogenetic tree and subfamily classification of GST proteins from sweet potato, *Ipomoea nil, Ipomoea trifida*, and *Arabidopsis thaliana*. The unrooted tree was constructed based on multiple sequence alignment of full-length protein sequences using Muscle program by MEGA 7.0 by the Maximum Likelihood (ML) method. In total, 42, 43, 74, and 55 full-length GST proteins from sweet potato, *I. nil*, *I. trifida*, and *A. thaliana*, respectively, were included. The percentage bootstrap scores which >50 are indicated on the nodes. The major phylogenetic subfamilies are illustrated as different color branches

Using phylogenetic analysis, we classified the 42 full-length *GST* proteins into seven major clades (i.e., subfamily): Tau (27 members), Phi (2), Theta (2), Zeta (3), EF1Bγ (3), Lambda (3), and DHAR (2) (Fig. 1a, Table 2, Additional file 5: Figure S2). A total of 10 putative motifs were predicted by the program MEME across all members. The arrangement of motifs within each subfamily was comparable, but diverse among different subfamilies (Fig. 1b). It has been reported that most of *GST* proteins contain motifs 1, 3, and 6, which jointly constitute the basis of N-terminal domain. Our analysis revealed a consistent result that the three motifs were present in almost all *GST* proteins. Motif 6 is found in members of Tau, Phi, Theta, EF1Bγ, and DHAR subfamilies; motifs 3 and 5 are specific to the Tau subfamily; Motif 7 is specific to EF1Bγ members; motif 8 and 9 are present in the Theta subfamily only; whereas motif 10 is found in the EF1Bγ and Theta subfamilies. We further analyzed the phylogenetic relationship among the *GST* proteins in sweet potato, *I. trifida, I. nil*, and *A. thaliana* (Additional file 6: Table S4). Figure 2 shows that 42 sweet potato GST proteins are clustered into eight subfamilies (Fig. 2 and Additional file 7: Figure S3) In all but TCHQD subfamilies, genes derived from each of the four species were included. In the TCHQD subfamily, no *GSTs* were identified in sweet potato and *I. trifida*. Overall, the majority of *GST* members of sweet potato, *I. trifida*, and *I. nil* were interspersed (i.e., show a mosaic pattern) within the phylogenetic tree, whereas most *A. thaliana* members were clustered into isolated subclades. These findings imply that most gene duplication events occurred probably before the speciation of sweet potato and after the divergence from *A. thaliana*.

**Table 2:** Number of different subfamilies of *GST* genes in nine species

GST gene subfamily	Tau	Phi	Theta	Zeta	EF1Bγ	Lambda	DHAR	TCHQD	Total
*Arabidopsis thaliana*	28	13	3	2	2	3	3	1	55
*Oryza sativa*	52	17	1	4	2	3	2	1	82
*Hordeum vulgare*	50	21	1	5	2	2	2	1	84
*Populus trichocarpa*	58	9	2	2	3	3	3	1	81
*Zea mays*	27	7	0	3	0	0	0	0	37
*Glycine max*	63	17	3	3	0	8	0	0	94
*Ipomoea trifida*	24	4	2	3	4	4	2	0	43
*Ipomoea nil*	48	6	4	7	4	3	2	1	74
*Ipomoea batatas*	27	3	2	3	3	3	2	0	43

Furthermore, we compared the number and distribution of *GST* genes across sweet potato and eight other plant species, including *Arabidopsis thaliana*, *Oryza sativa*, *Hordeum vulgare L.*, *Populus L.*, *Zea mays*, *Glycine max*, *Ipomoea trifida,* and *Ipomoea nil* (Table 2). The number of sweet potato *GST* genes (even plus 19 partial *GSTs*) identified in this study is less than those of *I. nil*, *O. sativa*, *H. vulgare, P. trichocarpa*, and *G. max*. This indicates that the collection of *GST* genes in our study was likely incomplete, and it might be hard to identify all members of a gene family from transcriptome databases. Amongst these, the Tau subfamily constitutes a biggest subfamily, which accounts for more than half of the total number of *GST* genes in each plant. In sweet potato, we identified 27 out of the 42 *GST* genes as Tau members. The Phi subfamily is the second-largest class of *GSTs* in various plants, and there are 13, 17, 21, 9, 7, and 17 in *Arabidopsis*, rice, barley, poplar, maize, and soybean. However, we identified that the Phi subfamily of sweet potato has only 3 members, which is less than the other species. Similarly, the number of Phi members in *I. trifida* and *I. nil* was 4 and 6, respectively. Although previous studies have shown that the Zeta and Theta subfamilies represent the oldest *GST* genes, their numbers in plants are less than those of plant specific subfamilies, Tau and Phi. These data suggest that members of the Tau and Phi subfamilies rapidly expanded in most plants and thus may play multifarious roles among various species.

**Fig. 3:**
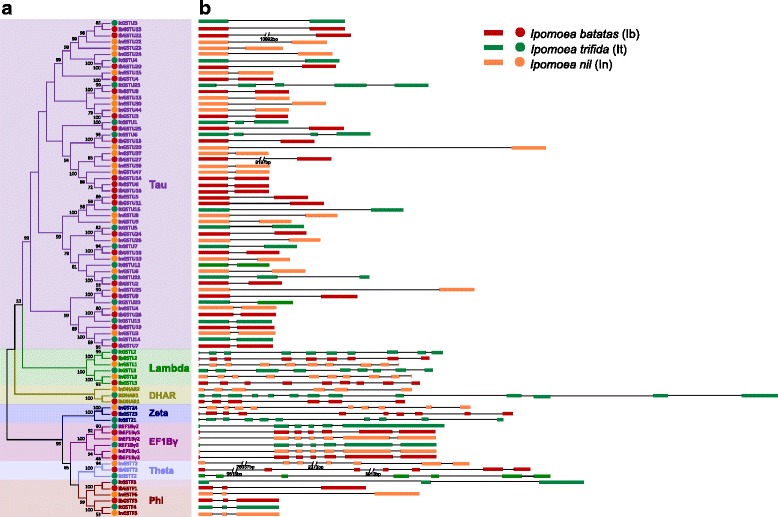
Phylogenetic relationships (**a**) and intron-exon structures (**b**) of homologous *GST* genes in sweet potato, *Ipomoea trifida*, and *Ipomoea nil*. In (**b**), filled boxes represent exons, whereas lines represent introns

To analyze the intron/exon structures of sweet potato *GST* genes, we aligned the coding sequences of 42 full-length *GST* genes against the published sweet potato genomic contig sequences [32]. We found corresponding genomic sequences for 30 sweet potato *GST* genes. Subsequently, we identified their best-matched homologous genes of *I. trifida* and *I. nil*, and compared their gene structures (Fig. 3). Our data indicated that the examined *GST* genes shared similar intron/exon structures within each subfamily but were differed among subfamilies. Most of the genes in the Tau subfamily possessed a single intron, whereas those in Zeta contained up to 10 introns. Nevertheless, exceptions were observed. For instance, *ItGSTU23* and *ItGSTU6* had 4 and 3 introns, respectively (Fig. 3b). Notably, we found some sweet potato genes containing unusually large introns, such as *IbGSTT2*. Comparative analysis of *IbGSTT2*, *InGSTT2*, and *ItGSTT2* indicated that each of these had 6 introns, whose sizes ranged from several hundreds to over 26,000 in base pairs (Fig. 3b). Taken together, our comparative analyses reinforce our viewpoint that most duplications and divergences of *GST* genes have likely occurred before the speciation of sweet potato (probably in a common ancestor of sweet potato, *I. trifida*, and *I. nil*).

### Gene expression profiles of the sweet potato *GSTs*

**Fig. 4:**
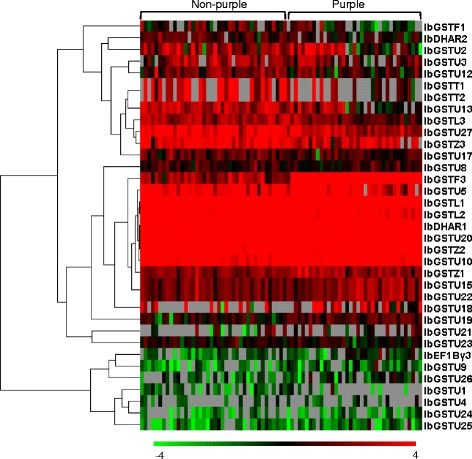
Hierarchical clustering of gene-expression data of 35 *GST* genes in 77 varieties of sweet potato. Horizontal direction indicates 77 sweet potato varieties, including 36 purple-flesh and 41 non-purple-flesh ones. The longitudinal direction indicates 30 *GST* genes in sweet potato. The data were FPKM values extracted from DB77 (log2 transformed) and color scaled as shown in the bottom. Gray boxes mean FPKM = 0

To investigate the diversification of expression patterns of sweet potato *GSTs*, we performed two RNA-seq experiments. First, we investigated variations in *GST* gene expression in mature tuberous roots of 77 sweet potato varieties. In total, we identified homologous transcripts in DB77 that represented 35 of our *GSTs*. FPKM values of homologous transcripts were extracted and used for a clustering analysis. Figure 4 shows differences in expression patterns in the examined *GSTs*: (1) some *GSTs* were highly expressed across most or all 77 varieties; (2) some genes were with extremely low expression in almost all varieties; and (3) some other genes exhibited variations in expression among different varieties. For example, *IbGSTL1*, *IbDHAR1*, *IbGSTU10*, *IbGSTU20*, and *IbGSTZ2* showed very high expression levels in all 77 sweet potato varieties, whereas *IbGSTU8*, *IbGSTU21*, and *IbGSTU23* exhibited low expression levels in almost all 77 varieties. These results indicate that the expression of each *GST* gene in the tuberous root is influenced by genetic background, which varies among sweet potato cultivars. Notably, *IbGSTF3* showed significantly higher expression in the tuberous roots of all purple-flesh sweet potato varieties than those in non-purple-flesh ones (Fig. 4, Fig. 7c).

**Fig. 5:**
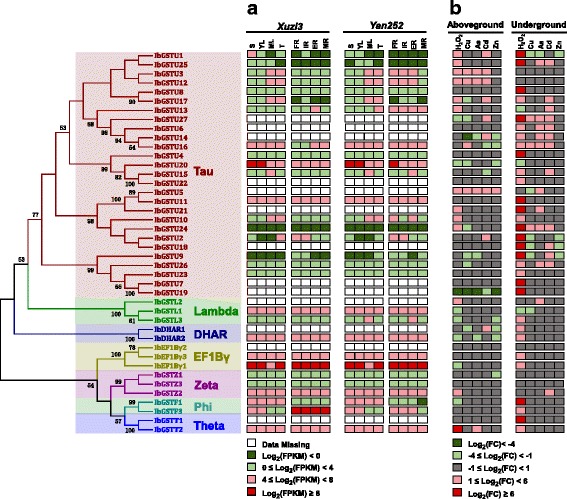
Gene-expression profiles of sweet potato *GSTs*. **a** The FPKM data of 27 *GST* genes across 8 different tissues of purple-flesh and non-purple-flesh sweet potato were extracted from DB16 (log2 transformed, see text for more details) and shown (dark green, log2(FPKM) < 0; light green, 0 < log2(FPKM) < 4; pink, 4 < log2(FPKM) < 8; red, log2(FPKM) > 8). DM, data missing; S, shoot; YL, young leaf; ML, mature leaf; T, stem; FR, fibrous root; IR, initial tuberous root; ER, expending tuberous root; MR, mature tuberous root, respectively. **b** Gene-expression profiles of sweet potato *GST* genes in response to various abiotic stresses. The fold changes (test to control samples in aboveground and underground tissues, respectively) of gene expression were calculated and shown. The expression data of all 42 full-length GSTs were obtained by quantitative RT-PCR. H_2_O_2_, hydrogen peroxide; Cu, copper; As, arsenic; Cd, cadmium; Zn, zinc, respectively

**Fig. 6:**
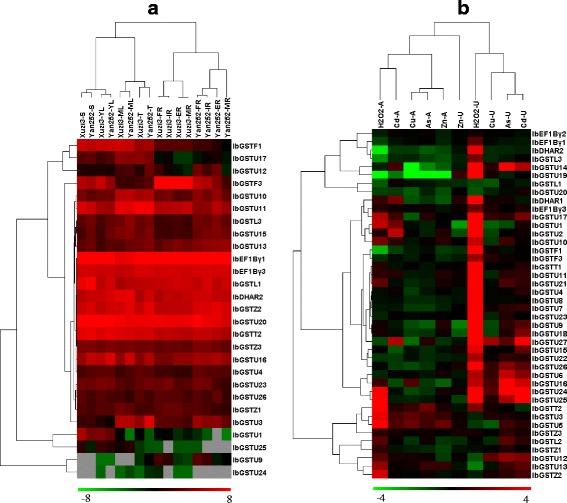
Hierarchical clustering of gene-expression data. **a** The FPKM data of 27 *GST* genes across 8 different tissues of purple-flesh and non-purple-flesh sweet potato were extracted from DB16 (log2 transformed, see text for more details) and color scaled as shown in the bottom (gray boxes mean FPKM = 0). S, shoot; YL, young leaf; ML, mature leaf; T, stem; FR, fibrous root; IR, initial tuberous roots; ER, expending tuberous root; MR, mature tuberous root. **b** The fold changes (test to control samples in aboveground and underground tissues, respectively) of gene expression in five experiments of abiotic stress treatments were calculated and color scaled as shown in the bottom. The expression data of all 42 full-length *GSTs* were obtained by quantitative RT-PCR. -A, aboveground; −U, underground; H_2_O_2_, hydrogen peroxide; Cu, copper stress; As, arsenic; Cd, cadmium; Zn, zinc

Second, we surveyed the expression patterns of *GST* genes in 8 different tissues of one purple-flesh and one non-purple-flesh sweet potato varieties. We assembled a transcriptome database (named as DB16) from all RNA-seq data of 16 samples and identified transcripts corresponding to 27 of our *GST* genes (Fig. 5a). Clustering analysis of the FPKM data revealed distinct expression patterns of these *GST* genes between aboveground and underground tissues. In the aboveground tissues, the gene expression data from two same tissues (e.g., *Xuzi3*-S and *Yan252*-S) of purple-flesh and non-purple-flesh sweet potato varieties were clustered together, whereas in the underground tissues, data from the four tissues of either purple-flesh or non-purple-flesh sweet potato were grouped together (Fig. 6a). These data suggest that substantial divergence in regulation of the *GST* genes has occurred between aboveground and underground tissues of sweet potato. Furthermore, some *GST* genes demonstrated highly specific expression patterns. For example, *IbGSTU1* showed relatively high expression only in shoot of purple-flesh sweet potato. On the other hand, we examined whether two phylogenetically close genes (i.e., with high similarity in coding sequences) exhibited highly mimic gene expression patterns across different tissues. We found that only a few phylogenetically close genes (e.g., *IbGSTU23*, and *IbGSTU26*) showed similar expression patterns, and the majority of genes did not (e.g., *IbGSTF1* and *IbGSTF3*, *IbGSTL1* and *IbGSTL3*) (Figs. 5a and 6a). These results imply that sequence divergence in coding sequences and regulatory regions of two duplicated genes was likely uncoupled.

**Fig. 7:**
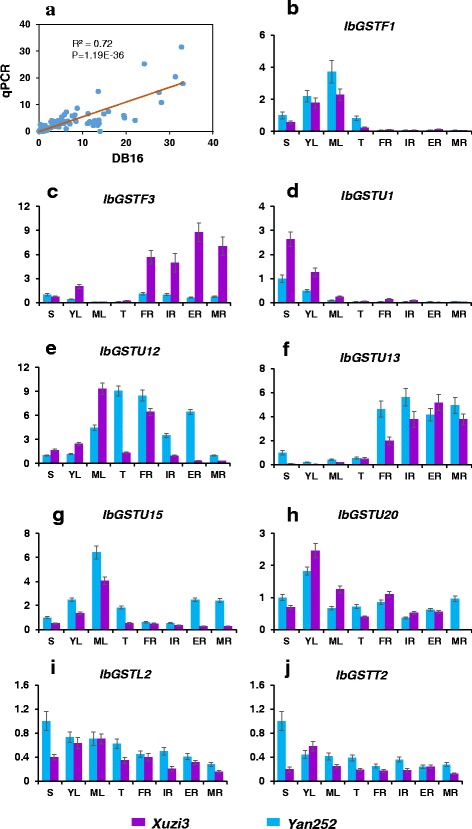
Quantitative PCR analysis of 9 *GST* genes to validate their expression profiles obtained from DB16. **a** The Pearson’s correlation between FPKM values obtained from DB16 and gene-expression data gained by qPCR. R^2^ and *P* value are shown. **b-j** Relative expression of 9 *GST* genes were examined in two varieties of sweet potato, one is purple-flesh (*Xuzi3*) and the other is non-purple-flesh sweet potato (*Yan252*). For each sample, three duplicate PCR reactions were performed and the resulting data were used to calculate the mean and the standard error, which are shown in the panels. S, shoot; YL, young leaf; ML, mature leaf; T, stem; FR, fibrous root; IR, initial tuberous root; ER, expending tuberous root; MR, mature tuberous root

In addition, we selected 9 *GST* genes and performed quantitative real-time PCR to confirm the gene expression patterns observed in the above described RNA-seq experiments. Overall, we found a high correlation in gene expression that was quantified using two approaches (Fig. 7a). That is, the expression patterns of 9 *GST* genes showed differences among varieties and tissues, which were in agreement with the data obtained by RNA-seq (Fig. 7b-j). These results demonstrate the high level of reliability of our gene expression data.

### Roles of sweet potato *GSTs* in response to abiotic stresses

Although sweet potato has the extraordinary ability to adapt to a wide spectrum of stresses, our understanding of its underlying molecular mechanisms is limited. Previous studies have revealed that *GST* genes are involved in plant responses to oxidative stresses and heavy metal toxicity [42, 43]. In the present study, we examined the expression patterns of 42 sweet potato *GST* genes in response to stress treatments using H_2_O_2_, Cu, As, Cd, and Zn, respectively. Gene expression data of each *GST* were compared between the treated and untreated tissues to infer a stress-response pattern. We found that the expression of all but two *GST* genes (*IbGSTZ3* and *IbEF1Bγ2*) significantly changed (i.e., more than two-fold increase or decrease) under at least one of the stress treatments (Fig. 5b). The significantly changed *GSTs* were found in each of seven subfamilies, which suggests that *GST* genes are widely involved in responses to diverse abiotic stresses in sweet potato. Moreover, hierarchical clustering analysis indicated that the overall stress-response patterns of investigated *GST* genes were remarkably different in the aboveground and underground tissues (Fig. 6b).

It is well-known that H_2_O_2_ plays dual roles in plant stress-response system, as a stressor that causes the injury to biological macromolecules and a signal molecule that induces the expression of a series of defense genes [44, 45]. The present study determined that with H_2_O_2_ treatment, the majority of *GST* genes were significantly upregulated (i.e., more than two-fold increase) in the underground tissues, whereas that of aboveground tissues varied (i.e., some were upregulated, some were downregulated, and some did not change (Figs. 5b and 6b). In particular, the most severely affected *GSTs* were found exclusively with H_2_O_2_ treatment [i.e., log_2_(fold changes) > 6; Fig. 5b]. These data indicate that a large number of sweet potato *GST* genes are involved and might play pivotal roles in stress-response pathways that are mediated or triggered by H_2_O_2_.

Arsenic and cadmium are two heavy metals that are usually toxic to plants. With arsenic and cadmium treatments, 12 and 14 *GSTs*, respectively, were significantly affected in the underground tissues. All but one gene were upregulated and 8 of these were common in both treatments, and most genes belong to the Tau subfamily (Fig. 5b). In contrast, distinct gene expression patterns were observed in the aboveground tissues (Fig. 5b). Copper and zinc are regarded as two necessary trace elements that cause stress when present at high concentrations in plants [46]. With copper treatment, a number of genes were significantly influenced, and most of the affected genes were downregulated. Two genes (*IbGSTU14* & *IbGSTU19*) were significantly downregulated in aboveground tissues (Fig. 5b). With zinc treatment, only one gene (*IbGSTU5*) was upregulated in the aboveground tissues; whereas several *GSTs* were downregulated, four in underground and nine in aboveground tissues (Fig. 5b).

Overall, the stress-response patterns of investigated *GST* genes substantially varied as a consequence of evolutionary diversification. Some *GST* genes are involved in the responses to multiple stressors, whereas some others respond to a specific stressor (Fig. 5b). In particular, in aboveground tissues, *IbGSTU19* was downregulated and *IbGSTU5* was upregulated in any treatment involving five abiotic stressors; *IbGSTU3* and *IbGSTU12* were upregulated after treatment with H_2_O_2_, Cu, As, and Cd. In underground tissues, *IbGSTU24*, *IbGSTU27*, and *IbGSTU16* were upregulated after treatment with H_2_O_2_, Cu, As, and Cd. Most of other *GSTs* responded to one or two stressors in either aboveground or underground tissues specifically. These results suggest that different *GST* members have been recruited and consequently divergent regulatory networks have been evolved in response to abiotic stresses in aboveground and underground tissues in sweet potato.

## Discussion

### Transcriptome-based gene family analyses in genetically complex organisms

Despite great advances in sequencing technologies, it remains costly and technically challenging to obtain high-quality reference genomes of genetically complex organisms [47, 48].In this study, we report 43 full-length and 19 partial *GST* genes that were identified from local transcriptome databases in sweet potato, a hexaploid crop lacking a high-quality reference genome. Molecular cloning and Sanger sequencing successfully validated the existence of 42 full-length *GST* genes (i.e., 97.67% correctness) in a single sweet potato variety. These data highlight the high quality of our transcriptome databases, which could be used for characterization of gene families. To our knowledge, this is the first study characterizing a gene family in sweet potato, which could be applied to other genetically complex organisms without currently available genomic sequences.

RNA-seq is not only useful for gene discovery but also for quantifying transcript abundance, which could be applied to study the spatiotemporal profile of a gene and the pattern of gene-expression divergence of a gene family. In the present study, we investigated the gene-expression profiles of 42 full-length *GST* genes in the tuberous roots of 77 sweet potato varieties (the DB77 dataset) and 8 different tissues of each of two varieties (the DB16 dataset). These experiments have provided fundamental gene expression data of *GST* genes and revealed important insights into the evolution (especially in regulatory regions) of the *GST* gene family. Today, it becomes costly affordable to survey a relatively large number of samples using RNA-seq and thus could be easily applied to genetically complex organisms.

Although transcriptome-based gene family analyses are feasible and useful in genetically complex organisms, one should be aware of its limitations and cautious in data interpretation. Firstly, it might be difficult to collect all members of a gene family in a species because only expressed members could be possibly gathered by RNA-seq. This could be the main reason why we identified relatively less *GSTs* out of DB12 and DB77 than those of the sweet potato relatives (e.g., *I. trifida* and *I. nil*; Table 2). Incomplete identification of gene family members might produce a wrong or incomplete phylogenetic tree and lead to imprecise interpretations. Second, transcriptome-derived sequences neither contain information on regulatory *cis*-elements nor exon-intron structures, which are important to infer the evolution of a gene family. Third, information on the locations of gene members on chromosomes is currently not available, which might hinder in investigations relating to genome evolution and speciation.

### Evolution and functional divergence of sweet potato *GST* genes

After gene duplication, mutations in two duplicates could occur in either coding sequences or regulatory regions. The former could modify protein properties and the latter might alter gene expression profiles spatiotemporally, both of which could result in functional divergence of duplicated genes [2, 49]. In the present study, we investigated the divergence of 42 full-length sweet potato *GSTs* in both coding sequences and gene-expression profiles. Our analyses revealed an evolutionary pattern for *GST* genes across various species: largely conversed within and highly divergent among gene subfamilies. These results suggest that the main gene subfamilies in sweet potato were of ancient origin. However, signatures specific to recent gene duplications within a subfamily (at least after species divergence of the ancestor of sweet potato from that of *A. thaliana*) were also detected. This is consistent to available knowledge that sweet potato underwent multiple whole genome duplication events during its hexaploidization [32]. However, how different homeologs within each subfamily diverged after sweet potato speciation remains unclear. On the other hand, we demonstrated that the expression of a specific *GST* gene was dependent on tissues as well as genetic backgrounds, and remarkable divergence had occurred in gene expression profiles among different *GST* paralogs, i.e., substantial genetic differentiations might have accumulated in regulatory regions of studied *GST* genes, even in members within the same subfamily.

The question whether diversification of coding sequences and gene expression patterns in duplicated genes are correlated has been a topic of intense debate [50]. Fox example, Wagner et al. (2000) studied the relationship between expression profiles and protein sequence among yeast duplicate genes and found no significant correlation [51]. Makova et al. (2003) uncovered that nonsynonymous (Ka) and synonymous (Ks) substitution rates were significantly correlated with gene expression divergences of human duplicate genes at early stages after duplication [50]. McCarthy et al. (2015) reported that the functional divergence between two *Arabidopsis* paralogous genes is attributable to both regulation and changes in coding sequence [52]. In our study, we examined whether two genes with high similarity in coding sequences shared high similarity in gene expression patterns across different tissues. We found that most closely related genes showed different gene expression patterns. Based on our results, we postulate that divergence in coding sequences and regulatory regions of the two paralogous *GST* genes is uncoupled.

### Distinct *GST*-mediated networks in aboveground and underground tissues of sweet potato in response to abiotic stresses

*GST* genes play important roles during plant growth, especially in plant defense or resistance to specific noxious chemicals. However, the function of each *GST* member, how the gene takes effect, and how the gene interplays each other remain unclear. In particular, related knowledge on the species sweet potato, a hexaploid crop with extraordinary capacity of adapting to different stressful environments such as high salinity, drought, and polluted soils, is limited. In the present study, we investigated the stress-response patterns of 42 sweet potato *GST* genes by the stress treatments of H_2_O_2_, Cu, As, Cd, and Zn, respectively. Our data clearly exhibited divergences in the stress-response patterns of *GST* paralogs, which is in agreement with our observations from sequence analyses and gene-expression profiles. The majority of *GST* genes were specifically involved in response to one or two single stressors, whereas some *GSTs* responded to multiple abiotic stresses (e.g., *IbGSTU5*, *IbGSTU19*, *IbGSTU24*, and *IbGSTU27*). These results imply that the biological functions as well as the degree of importance of different *GST* paralogs were highly divergent in the whole stress-response system in sweet potato and likely in other higher plants. In particular, almost all investigated *GSTs* showed distinct stress-response patterns between aboveground and underground tissues (Fig. 5b). Based on our results, we inferred that different *GST*-mediated networks involved in aboveground and underground tissues in response to abiotic stresses in sweet potato. In underground tissues, abiotic stresses caused by heavy metals and/or oxidizing agents (e.g., H2O2) would trigger signals, which subsequently activate specific *GST* genes (e.g., *IbGST24*, *IbGSTU27*, and *IbGSTU16*). Meanwhile, the signals would be transmitted to aboveground tissues where some other *GST* genes were activated (e.g., *IbGSTU5* and *IbGSTU12*) or repressed (e.g., *IbGSTU19*). Further studies on how different *GST* members coordinate or interplay in the whole stress-response system are thus warranted.

## Conclusions

In this study, we demonstrates the first example of transcriptome-based gene family analyses in sweet potato, a genetically complex and agronomically important crop. We identified and comparatively analyzed 42 full-length sweet potato GSTs in both coding sequences and gene-expression profiles, as well as their stress-response patterns. Our study systematically investigated the diversification of *GST* genes in sweet potato and provides useful information for elaborating the *GST*-mediated stress-response system in this worldwide crop as well as other plants.

## Additional files

Additional file 1: Figure S1. Tissue sampling for DB12, DB16, and DB77. (DOCX 1990 kb)

Additional file 2: Table S1. Primers used in this study. (XLSX 15 kb)

Additional file 3: Table S2. Information of sweet potato GST genes identified in this study. (XLSX 59 kb)

Additional file 4: Table S3. Pairwise identity matrix for full-length GST genes in this study. (XLSX 22 kb)

Additional file 5: Figure S2. Phylogenetic relationships of 42 sweet potato GST proteins. (DOCX 41 kb)

Additional file 6: Table S4. Information of GSTs from other species used in this study. (XLSX 39 kb)

Additional file 7: Figure S3. Phylogenetic tree and subfamily classification of GST proteins from sweet potato, *Ipomoea nil*, *Ipomoea trifida*, and *Arabidopsis thaliana*. (DOCX 1419 kb)

## Abbreviations

FPKM: Fragments per kilobase of transcript per million fragments mapped
GST: Glutathione S-transferase
RNA-seq: whole transcriptome shotgun sequencing
