# Supplementary material for: Identification and analysis of glutathione S-transferase gene family in sweet potato reveal divergent GST-mediated networks in aboveground and underground tissues in response to abiotic stresses
Source: BMC Plant Biol. 2017 Nov 28;17:225. doi: 10.1186/s12870-017-1179-z (PMC5704550; doi:10.1186/s12870-017-1179-z)
Supplement: Supplementary file 1 — Tissue sampling for DB12, DB16, and DB77. (DOCX 1990 kb) [file 12870_2017_1179_MOESM1_ESM.docx]

**Additional file 1. Figure S1**


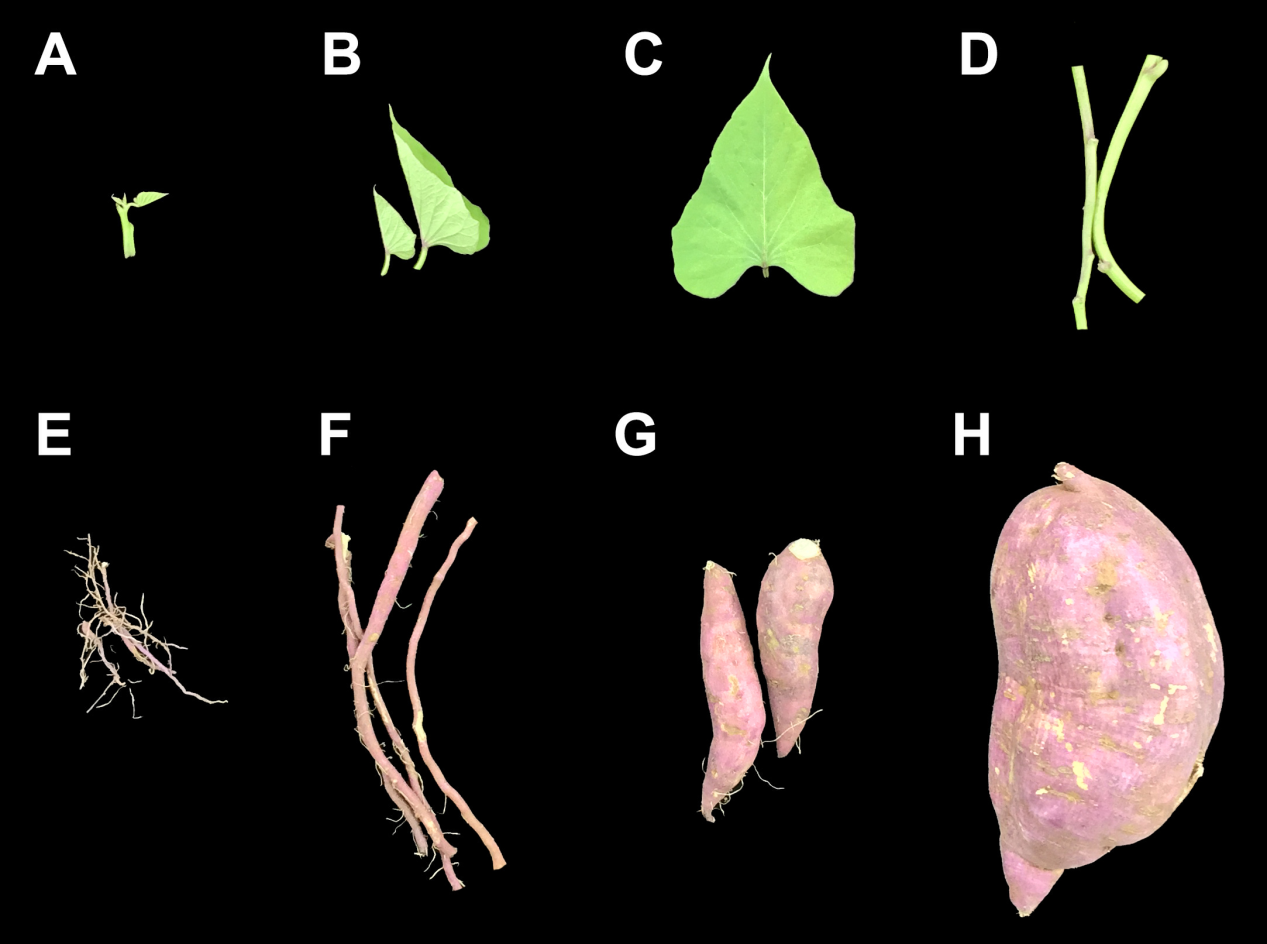


**Figure S1. Tissue sampling for DB12, DB16, and DB77.** For DB12, *Xushu18* (one of the most widely cultivated sweet potato varieties in China) was selected for the experiments. Tissues of shoots (**A**), young leaves (**B**), mature leaves (**C**), stems (**D**), fibrous roots (**E**), initiating tuberous roots (**F**), expanding tuberous roots (**G**), and mature tuberous roots (**G**) were collected and pooled together in approximately equivalent weights for extraction of total RNA. For DB16, the same 8 tissues were harvested, separately, from one purple-flesh (*Xuzi3*) and one non-purple-flesh (*Yan252*) sweet potato variety. For DB77, tissues of mature tuberous roots (**G**) were collected, separately, from each of 77 varieties and used for RNA sequencing.
