# Supplementary material for: Identification and analysis of glutathione S-transferase gene family in sweet potato reveal divergent GST-mediated networks in aboveground and underground tissues in response to abiotic stresses
Source: BMC Plant Biol. 2017 Nov 28;17:225. doi: 10.1186/s12870-017-1179-z (PMC5704550; doi:10.1186/s12870-017-1179-z)
Supplement: Supplementary file 5 — Phylogenetic relationships of 42 sweet potato GST proteins. (DOCX 41 kb) [file 12870_2017_1179_MOESM5_ESM.docx]

**Additional file 5. Figure S2**

**Figure S2. Phylogenetic relationships of 42 sweet potato GST proteins.** Multiple alignments of amino acids of 42 full-length *GST* genes from sweet potato were executed by Muscle Program and the phylogenetic tree was constructed using MEGA 7.0 by the Neighbor-joining (NJ) method.
