# Supplementary material for: Identification and analysis of glutathione S-transferase gene family in sweet potato reveal divergent GST-mediated networks in aboveground and underground tissues in response to abiotic stresses
Source: BMC Plant Biol. 2017 Nov 28;17:225. doi: 10.1186/s12870-017-1179-z (PMC5704550; doi:10.1186/s12870-017-1179-z)
Supplement: Supplementary file 7 — Phylogenetic tree and subfamily classification of GST proteins from sweet potato, Ipomoea nil, Ipomoea trifida, and Arabidopsis thaliana. (DOCX 1419 kb) [file 12870_2017_1179_MOESM7_ESM.docx]

**Additional file 7. Figure S3**

**Figure S3. Phylogenetic tree and subfamily classification of GST proteins from sweet potato, *Ipomoea nil, Ipomoea trifida*, and *Arabidopsis thaliana*.** Multiple alignments of amino acids were executed by Muscle Program and the phylogenetic tree was constructed using MEGA 7.0 by the Neighbor-joining (NJ) method with 1,000 bootstrap replicates.
